# Identification of causal effects on binary outcomes using structural mean models

**DOI:** 10.1093/biostatistics/kxq024

**Published:** 2010-06-03

**Authors:** Paul S. Clarke, Frank Windmeijer

**Affiliations:** Centre for Market & Public Organisation, University of Bristol, 2 Priory Road, Bristol BS8 1TX, UK paul.clarke@bristol.ac.uk; Department of Economics and Centre for Market & Public Organisation, University of Bristol, 8 Woodland Road, Bristol BS8 1TN, UK

**Keywords:** Causal inference, Complier average causal effects, Instrumental variables, Local average treatment effects, Principal stratification

## Abstract

Structural mean models (SMMs) were originally formulated to estimate causal effects among those selecting treatment in randomized controlled trials affected by nonignorable noncompliance. It has already been established that SMMs can identify these causal effects in randomized placebo-controlled trials under fairly weak assumptions. SMMs are now being used to analyze other types of study where identification depends on a *no effect modification* assumption. We highlight how this assumption depends crucially on the unknown causal model that generated the data, and so is difficult to justify. However, we also highlight that, if treatment selection is monotonic, additive and multiplicative SMMs do identify local (or complier) causal effects, but that the double-logistic SMM estimator does not without further assumptions. We clarify the proper interpretation of inferences from SMMs by means of an application and a simulation study.

## 1. INTRODUCTION

Robins (1989, 1994) introduced the class of semiparametric structural mean models (SMMs) and “G-estimation” for inferences about the causal effects of treatment regimes on outcomes from randomized controlled trials affected by noncompliance. Noncompliance comes about in encouragement designs where participants can choose treatments other than those to which they were randomized. Of most interest are SMM estimators that allow for the effects of nonignorable noncompliance, that is, where participants choose their treatments in a manner associated with their study outcomes, even after baseline (and possibly time-varying) covariates have been adjusted for. We note that alternative approaches for obtaining causal inferences have also been proposed (e.g., Goetghebeur *and others*, 1998).

The parameters of SMMs correspond to meaningful functions of expected potential outcomes for the population of participants exposed to the treatment. For example, additive SMMs are specified in terms of average treatment (or causal) effects, and multiplicative SMMs in terms of causal risk ratios. Vansteelandt and Goetghebeur (2003) developed the generalized SMM and we consider the important special case of the logistic SMM and its “double-logistic” estimator for causal odds ratios (OR). Hernan and Robins (2006) review additive and multiplicative SMMs and consider the relationship with econometric instrumental variable estimators; Goetghebeur and Vansteelandt (2005) review all of the SMMs considered here.

In this paper, we consider the estimation of causal effects using SMMs from studies with binary outcomes. More precisely, we consider the conditions under which each SMM estimator identifies its target causal parameter, and the consequences arising if these conditions do not hold. Until recently, generalized SMMs had mainly been applied to randomized placebo-controlled trials, for which the identification issue is fairly straightforward. However, SMMs can be applied to other types of randomized controlled trial, and more generally to the causal analysis of observational studies using instrumental variables. For these designs, Hernan and Robins (2006) show that the usual identification assumption is “no effect modification by randomisation” (NEM). We highlight how identification depends crucially on whether the unknown data generating process satisfies the strong constraints imposed by NEM.

Another question we address is: what causal parameter is being identified if NEM does not hold? As such, we highlight previous results showing that additive and multiplicative SMMs identify “local,” or “complier,” causal effects under the alternative assumption that patients' treatment selection is monotonic (e.g., Angrist *and others*, 1996; Frangakis and Rubin, 2002). However, we also highlight that the double-logistic SMM does not identify the local odds ratio (LOR) under monotonic selection, and that an alternative estimator for the LOR must be used instead.

The remainder of this paper is organized as follows. In Section 2, we review the potential outcomes causal framework within which SMMs are specified, and the three important SMMs considered in this paper. In Section 3, we consider the identification of each SMM's causal effect for randomized placebo-controlled trial designs, before going on in Section 4 to consider identification for more general designs. An alternative identification strategy based on monotonic treatment selection is considered in Section 5. Finally, in Section 6, we consider a data example and present some numerical results to illustrate the potential impact on results if NEM does not hold, before making our concluding remarks in Section 7.

## 2. STRUCTURAL MEAN MODELS

### 2.1 Potential outcomes

Before introducing SMMs, we first set out the potential outcomes notation to be used throughout. To simplify notation and highlight concepts, we consider only the simplest setup: a randomized controlled trial with an encouragement design in which patients are randomized to a fixed treatment dose or to the control group, which they comply with or not according to some nonignorable mechanism; the binary study outcome is measured after some fixed follow-up period. The focus on this simple setup is done without loss of generality, and our findings apply equally to situations including prerandomization covariates, variable treatment dose, and treatment regimes involving repeated doses with time-varying covariates recorded.

Following Hernan and Robins (2006), let *Y*,*X* and *Z* denote random variables representing the following observed quantities: *Z* is the randomization assignment indicator, with *Z* = 1 denoting treatment and *Z* = 0 control; *X*∈{0,1} is the corresponding indicator for the actual treatment chosen by the patient, where *X*≠*Z* is possible due to noncompliance; and *Y*∈{0,1} is the binary study outcome. It is assumed throughout that the observed data {(*y*_*i*_,*x*_*i*_,*z*_*i*_):*i* = 1,...,*n*} are i.i.d. realizations.

The potential outcomes can now be defined in the usual way. Let *Y*(*x*,*z*) be the potential outcome resulting from the treatment assignment being set to *z* and the treatment received to *x* by external intervention, rather than by the trial process in which the patient chooses treatment following randomization. Similarly, let *X*(*z*) be the potential treatment the patient would choose if the treatment assignment was set to *z* by external intervention.

Five important conditions for the identification of causal effects can now be stated as follows: the “stable unit treatment value assumption” that each patient's potential outcomes are unrelated to those of any other patient; the existence of “causal effects” of *Z* on *X*; the “consistency assumption” *Y* = *Y*(*X*,*Z*) and *X* = *X*(*Z*), linking the observed and potential outcomes; the “exclusion restriction” *Y*(*x*,*z*) = *Y*(*x*) constraining the effect of treatment assignment to affect the study outcome *only* through its effect on treatment choice; and the “independence assumption” implying that *Z* is independent of the potential treatments and outcomes {*X*(0),*X*(1),*Y*(0),*Y*(1)} (e.g., Angrist *and others*, 1996; Robins and Rotnitzky, 2004).

More generally, *Z* can be any instrumental variable (IV) satisfying these assumptions. The scope of application for SMMs is thus far broader than just randomized controlled trials, and encompasses observational studies too. However, it can be very difficult to find plausible candidates for *Z* because not all of the conditions above can be justified on empirical grounds alone. Hence, to maintain focus, all these assumptions will be taken to hold throughout this paper, and so we assume that a valid IV is available to the analyst.

### 2.2 The additive and multiplicative SMMs

For the simple scenario just described, the additive SMM is:
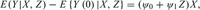
where *Y*(0) is the treatment-free potential outcome. While this model is saturated or nonparametric, more generally the right hand side is a parametric function incorporating the effect of prerandomization covariates or variable treatment dose. The parameters of the additive model are *ψ*_0_ = *E*{*Y*(1) − *Y*(0)|*X* = 1,*Z* = 0} and *ψ*_0_ + *ψ*_1_ = *E*{*Y*(1) − *Y*(0)|*X* = 1,*Z* = 1}, that is, the average treatment effect among those who choose treatment but were assigned the control, and the effect among those who are assigned to and chose treatment, respectively.

SMM estimators work by exploiting the conditional mean independence (CMI), or randomization, assumption:(2.1)
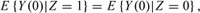
which follows from the conditions in Section 2.1. Under the additive SMM, (2.1) can be rewritten as(2.2)
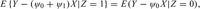
from which an estimating equation can be constructed.

The saturated multiplicative SMM for the same scenario is defined as:
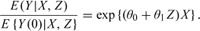
The parameters of the multiplicative SMM are the causal risk ratios among the same two subgroups as before. Under the multiplicative SMM, the CMI assumption (2.1) leads to the moment condition:(2.3)
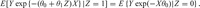



It is clear that neither set of SMM parameters is identified by its corresponding moment condition because both constitute systems with two unknowns and one equation. Therefore, further assumptions are required to identify the SMM parameters. Hernan and Robins (2006) highlight the role of the no effect modification (NEM) by *Z* assumption. Each SMM has its own distinct NEM assumption: for the additive SMM, it corresponds to constraining *ψ*_1_ = 0, and for the multiplicative SMM it corresponds to *θ*_1_ = 0. Under NEM, there is only one unknown in (2.2) and (2.3) and the usual target parameters are identified: for the additive SMM, the target becomes the average treatment effect among the treated *ψ*_0_ = *E*{*Y*(1) − *Y*(0)|*X* = 1}; for the multiplicative SMM, it is the causal risk ratio among the treated exp(*θ*_0_) = *E*{*Y*(1)|*X* = 1}/*E*{*Y*(0)|*X* = 1}.

The estimators of the additive and multiplicative SMM target parameters can be written as(2.4)
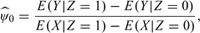
and(2.5)
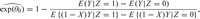
respectively (e.g., Angrist, 2001; Hernan and Robins, 2006). The additive SMM estimator has the same form as the classical instrumental variable estimator (Angrist *and others*, 1996); the numerator in both expressions is the intention to treat estimator. More generally, the estimating equations under additive and multiplicative SMMs based on (2.1) can be solved by G-estimation (Robins, 1994). For example, a multiplicative SMM G-estimator has been proposed for trials involving repeated binary outcomes and exposure measures (Robins, Greenland, *and others*, 1999).

### 2.3 The double-logistic SMM

Robins, Rotnitzky, *and others* (1999) proposed the logistic SMM with parameters corresponding to causal OR among the treated for the same groups as above. The logistic SMM can be written as
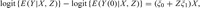
where logit(*a*) = log{*a*/(1 − *a*)} and NEM corresponds to the assumption that *ξ*_1_ = 0. Under NEM, the parameter exp(*ξ*_0_) is not quite equal to the causal OR among the treated because of noncollapsibility, but it can be viewed as a useful approximation.

The logistic SMM is considered separately here because it has been shown that no G-estimator exists for *ξ*_0_ (e.g., Robins and Rotnitzky, 2004). Vansteelandt and Goetghebeur (2003) developed the double-logistic estimator by exploiting the result that *ξ*_0_ can potentially be identified if the researcher specifies a parametric “association model” *E*(*Y*|*X*,*Z*) = *m*_**η**_(*X*,*Z*), which is indexed by parameter vector **η**. The double-logistic estimator is based on specifying *m*_**η**_(*X*,*Z*) to be logistic; the resulting moment condition is:(2.6)
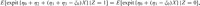
where expit(*a*) = exp(*a*)/{1 + exp(*a*)}. In practice, an estimate of **η** = (*η*_0_,*η*_1_,*η*_2_,*η*_3_)^′^ is obtained at the first stage by fitting the saturated logistic association model *m*_**η**_(*X*,*Z*) = expit(*η*_0_ + *Z* *η*_1_ + *X* *η*_2_ + *Z* *X* *η*_3_) and then substituted into (2.6). Vansteelandt *and others* (2010) consider issues arising when the logistic specification of both the SMM and the association model is uncongenial, but this poses no problem for the simple logistic SMM considered here.

## 3. THE NO CONTAMINATION RESTRICTION

There is a wide scope for applications of SMMs to randomized placebo-controlled trial designs, such as those considered by Greenland (2000), Nagelkerke *and others* (2000) and Vansteelandt and Goetghebeur (2003). In these designs, neither compliers nor noncompliers randomized to control can receive the treatment because noncompliers (*Z* = 0,*X* = 1) receive only the placebo, equating to the condition Pr(*X* = 0|*Z* = 0) = 1. Cuzick *and others* (2007) refer to this as a “no contamination” restriction, which is a special case of the identifying assumptions for binary outcome SMMs described by Robins and Rotnitzky (2004). To analyse placebo-control designs, an additional assumption of no placebo effect is also needed that we herein take to hold.

Under the no contamination restriction, the SMM parameters *ψ*_0_, *θ*_0_ and *ξ*_0_ are not defined because all three are conditioned on the measure-zero event {*X* = 1,*Z* = 0}. Conversely, {*X* = 1} = {*X* = 1,*Z* = 1} and so *ψ*≡*ψ*_0_ + *ψ*_1_ is the average treatment effect among the treated, exp(*θ*)≡exp(*θ*_0_ + *θ*_1_) is the causal risk ratio among the treated, and exp(*ξ*)≡exp(*ξ*_0_ + *ξ*_1_) is the causal OR among the treated. It follows that these parameters are all identified because it can be shown that the crucial *E*{*Y*(0)|*Z* = 0} is equal to *E*(*Y*|*Z* = 0) (e.g., Clarke and Windmeijer, 2010, section 3).

## 4. THE NEM ASSUMPTION

The role of NEM becomes crucial for more general designs. Each SMM has its own distinct NEM assumption, which acts to constrain the causal effects among the treated to be equal for those randomized to treatment and those randomized to control. To take just one example, recall that the additive NEM assumption constrains *ψ*_1_ = 0 in the additive SMM, and thus
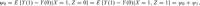
The NEM assumptions for the multiplicative and logistic SMMs can be similarly expressed.

To investigate the validity of NEM for binary outcomes, we make a link between structural models and potential outcomes by following Hernan and Robins (2006) and, less directly, Pearl (2000). Suppose that the analyst is faced with data from a randomized controlled trial for which the no contamination restriction does not hold. In an application, the observed data and all the counterfactual potential outcomes and potential treatments are realizations from an unknown “nonparametric structural equation model” that satisfies CMI and the constraints set out in Section 2.1 (noting that nonparametric here does not imply that the true data generating process cannot be parametric, only that no constraints are placed on its unknown form).

Using this link, the potential outcome can be written
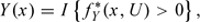
where indicator function *I*(*a*) = 1 if *a* is true and 0 otherwise, and *f*_Y_^*^(*x*,*U*) is a function that depends on the fixed value of treatment and the latent random variable/vector *U*. It is usual to interpret *U* as the combined effect of all unobserved confounding variables on the outcome, although it also involves the contributions from other variables which are independent of the exposure selection mechanism; to ensure independence of *Z* and *Y*(*x*) it follows that *U* must be independent of *Z*. The potential treatment is similarly defined as *X*(*z*) = *I*{*f*_X_^*^(*z*,*V*) > 0}, where *V* is another latent random variable/vector representing unobservable factors influencing treatment choice; it follows that *V* and *Z* must be independent.

If *U* and *V* are independent then noncompliance is ignorable, otherwise it is nonignorable. For fixed *x*, it is *U* that determines whether the potential outcome is zero or one for a particular patient, with *z* and *V* playing the same role for *X*(*z*). This setup straightforwardly extends to continuous potential treatments by dropping the indicator function and specifying *X*(*z*) = *f*_X_^*^(*z*,*V*) (see Clarke and Windmeijer (2010), appendix).

This class of structural models is extremely general because *f*_Y_^*^(*x*,*u*) can be any function generating, for example, nonlinear or heterogeneous treatment effects. However, it does not include models where the joint support of *U* and *V* depends on other variables. For example, models like *Y*(*x*) = *f*_Y_^*^(*x*) + *U* and *Y*(*x*) = *f*_Y_^*^(*x*)*U*, for which *Y* = *f*_Y_^*^(*X*) + *U* and *Y* = *f*_Y_^*^(*X*)*U*, respectively, are excluded because in both cases the support of *U* clearly depends on *X* to ensure that the outcome lies in {0,1}. These models are also structurally implausible because the support of *U* depends on its causal antecedent *X*.

Crucially, all the SMM parameters are functions of *E*{*Y*(*x*)|*X*,*Z*} and so can be written in terms of the underlying structural model as:
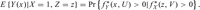
An advantage of defining the class of models in this way is that all its members satisfy the CMI assumption, which can be shown by expanding (2.1) and using the identity Pr{*f*_X_^*^(*z*,*V*) > 0} = *E*(*X*|*Z* = *z*). We can therefore focus on each of the NEM assumptions. For a specific example, consider the family of simple parametric structural models with (*U*,*V*) a bivariate continuous random vector related to the potential outcomes by(4.1)
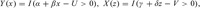
where *E*(*U*) = *E*(*V*) = 0 and (*U*,*V*) has distribution function *F*_*ρ*_(*u*,*v*), with “correlation” parameter *ρ* indexing all nonzero moments involving products of *U*_*k*_ and *V*_*k*_(*k* = 1,2,.....). In this case,
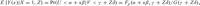
where *G*(*v*) is the marginal distribution function for *V*. Clearly, if noncompliance is ignorable, then *ρ* = 0, and all three NEM assumptions automatically hold. However, if *ρ*≠0, then none of the NEM assumptions will necessarily hold. To see why, consider the “bivariate probit model” where (4.1) holds, and *F* is the distribution function for a zero-mean, unit-variance bivariate normal distribution. If the data were generated from a model closely approximated by this, then NEM cannot hold for the additive, multiplicative or the logistic SMM because, for example,
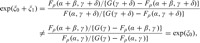
almost everywhere. As such, none of the SMMs can identify the associated causal parameters *ψ*_0_, exp(*θ*_0_) or exp(*ξ*_0_).

The bivariate probit example, for which NEM fails, is quite restrictive, but the class of all structural models is broader even than the family of models defined by (4.1). In practice, a data set may have been generated by a structural model that allows nonlinear effects of treatment and IV on the latent scale, or multivariate latent variables with semicontinuous or discrete distributions, or any combination of these features. The problem is that NEM places strong restrictions on the class of structural models, and it is impossible to verify whether or not these restrictions hold.

To illustrate the restrictions imposed by NEM, we focus on the logistic SMM and its NEM assumption. It is virtually impossible to write down this model in the same way as for the bivariate probit, but data can be generated which satisfy both the logistic SMM and its NEM assumption without specifying the underlying model (Robins and Rotnitzky, 2004; Vansteelandt *and others*, 2010). Data are generated as follows: first, generate *X* as Bernoulli with success probability, *E*(*X*|*Z*) = expit(*γ* + *δ* *Z*); second, generate the treatment-free outcomes as Bernoulli with success probability, *E*{*Y*(0)|*X*,*Z*} = expit(*β*_0_ + *β*_1_ *X* + *β*_2_ *Z*); and finally, generate observed outcomes using success probability, *E*(*Y*|*X*,*Z*) = expit{*β*_0_ + (*β*_1_ + *ξ*_0_)*X* + *β*_2_ *Z*}, where *ξ*_0_ is the target parameter. Clearly this model satisfies the NEM assumption *ξ*_1_ = 0, but to identify *ξ*_0_, we additionally require that CMI holds and so must constrain the *β*-parameters to satisfy
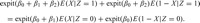



An example of a model satisfying this data generating process can be written as:
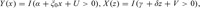
where *U* = (*β*_0_ − *α*) + *β*_1_ *X* + *β*_2_ *Z* + *W* and (*V*,*W*) has standard logistic marginal distributions that are both mutually independent and independent of *Z*. This model does not fit into the structural setup defined above because *U* and *V* are associated only indirectly through *X* and *Z*, and *U* is clearly not independent of *Z*. However, it does show how restrictive the family of models satisfying the logistic NEM is: the integral of *Y* = *I*{*f*_Y_^*^(*X*,*U*) > 0} with respect to the conditional distribution of *U* given *X* and *Z* (and an appropriate measure) must be a logistic function, whereas the class of structural models we consider places no such restriction. The existence of other families is not merely a theoretical artifact: the probit SMM (Goetghebeur and Vansteelandt, 2005) is often considered plausible but does not satisfy NEM for the logistic SMM. Clarke and Windmeijer (Appendix 2010) discuss this issue in more detail.

## 5. MONOTONIC SELECTION

We have argued that the families of models for binary outcomes and treatments satisfying the additive, multiplicative, or logistic NEM assumptions are very restrictive, and establishing NEM holds (even approximately) depends on application-specific background knowledge that is extremely difficult—if not impossible—to obtain. As such, we now discuss an alternative assumption to NEM, namely, monotonic selection of treatment by patients, under which local, or complier, causal effects can be estimated. Angrist *and others* (1996) highlight the importance of “monotonicity.” where patients' selection of treatment is monotonic if(5.1)
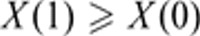
for some coding of *X*,*Z*; in general, selection is monotonic if *z* > *z* ^′^⇒*X*(*z*) ≥ *X*(*z* ^′^) (Imbens and Angrist, 1994). For example, the simple structural model described in Section 4 is monotonic because *X*(1) = *I*(*γ* + *δ* − *V* > 0) ≥ *I*(*γ* − *V* > 0) = *X*(0) if *δ* > 0.

In this setup, monotonic selection corresponds to the assumption that no patient will be a “defier” (i.e., one where *X*(0) = 1,*X*(1) = 0) with probability 1. Conversely, patients may be compliers (*X*(0) = 0,*X*(1) = 1 or *X*(1) > *X*(0)), “always-takers” (*X*(0) = *X*(1) = 1) or “never-takers” (*X*(0) = *X*(1) = 0). For these definitions to make sense, we can assume that all patients exist in two universes, one in which they are randomized to control, and another in which they are randomized to treatment. Hence, the “no defiers” assumption corresponds to saying that, while patients can disobey their treatment assignments in one or other of these universes, they cannot disobey their assignments in both. Frangakis and Rubin (2002) refer to the stratification of patients using intermediate outcomes in this way (treatment *X* in this example) as “principal stratification.”

While the NEM assumption does not generally hold, additive and multiplicative SMM estimators (2.4) and (2.5) do identify local effects under monotonic selection. Specifically, consider estimator (2.4) based on the additive SMM. As noted previously, it has the same form as the classical instrumental variable estimator, and so from the results of Imbens and Angrist (1994) it follows that it is consistent for the local average treatment effect LATE = *E*{*Y*(1) − *Y*(0)|*X*(1) > *X*(0)}, which is also called the “complier average causal effect.” (Note that no contamination can be seen as an extreme special case of monotonic selection in which *X*(1) ≥ *X*(0) = 0 and the complier and treated groups are equivalent.) Similarly, Angrist (2001) shows that estimator (2.5) based on the multiplicative SMM under NEM is consistent for the local relative risk (LRR), which is defined as:
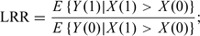
see also Greenland (2000) and Hernan and Robins (2006).

The LOR is defined in a similar way to the LRR. Our numerical examples below illustrate that the double-logistic estimator based on (2.6) is biased for the LOR under monotonic selection. Clarke and Windmeijer (appendix 3 2009) show that the double-logistic estimator is not consistent for the LOR under monotonic selection unless *E*{*Y*(1)|*X*(1) = *X*(0) = 1} = *E*{*Y*(1)|*X*(1) > *X*(0)}. Abadie (2003) proposes a consistent estimator for the LOR. As noted by van der Laan *and others* (2007), an estimator can also be based on (2.5): first calculate 
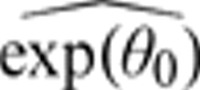
 as per usual, then recode the outcome variable as *Y* ^*^ = 1 − *Y* and calculate 
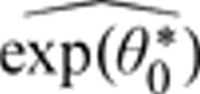
 replacing *Y* by *Y* ^*^ in (2.5); the ratio 
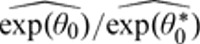
 is consistent for the LOR by symmetry of the relative risk (RR). We herein refer to this as the “LOR estimator”.

## 6. NUMERICAL EXAMPLES

### 6.1 Data example

To summarize and make the implications of these results concrete, consider the following example of an observational study to which instrumental variables have been used to obtain causal inferences. A study of patients attending clinical practice was carried out to assess if the “Cox-2” inhibitor treatment performed better than the standard, nonselective nonsteroidal anti-inflammatory (NSAID) treatment in preventing the unwanted side effect of gastrointestinal bleeding after 60-day follow-up (Brookhart *and others*, 2006). The analysis here is based on a subset of 37 842 patients who took part in the original study, of which 26 407 were allocated Cox-2 and 11 435 allocated NSAID by their physicians (Brookhart and Schneeweiss, 2007; Vansteelandt *and others*, 2010).

In our setup, *Y* is 1 if the patient experiences gastrointestinal bleeding within 60 days of being treated and 0 otherwise; and *X* is 1 if the patient receives Cox-2, and 0 otherwise. The IV*Z* for each patient is taken to be the treatment allocated by the prescribing physician to the preceding patient. Brookhart *and others* (2006) originally proposed the use of physician preference for this study. We take *Z* to be a valid IV and refer the reader to Hernan and Robins (2006) for a detailed discussion of how well physician preference satisfies the conditions set out in Section 2.1.

We fit the additive, multiplicative, and logistic SMMs to these data, along with the naive logistic regression of *Y* on *X*, and estimate the LOR as described above; two-tailed 95% percentile confidence intervals (CI) are also calculated based on 100 nonparametric bootstrap samples. The results are displayed in Table 1. The naive OR based on the logistic model is 1.032, indicating a negative effect of Cox-2 over NSAIDs in the trial; the CI is (0.80 − 1.37) and includes 1, which indicates that there is insufficient evidence to reject the hypothesis that the treatments are the same.

**Table 1.** Logistic regression, additive SMM, multiplicative SMM, LOR, and double-logistic SMM estimates from an encouragement trial design comparing incidence of gastrointestinal bleeding in patients choosing Cox-2 inhibitors compared to those choosing NSAIDs (Brookhart and others, 2006)

Estimator	Estimate	95% CI
Logistic OR	1.032	0.80 – 1.37
Add. SMM	−0.0092	−0.017to – 0.002
Mult. SMM	− 0.176	− 1.56 to 0.81
LOR Estimator	−0.174	−1.56to 0.70
DL SMM	0.029	0.01 – 0.73

95% CI calculated from 100 bootstrap samples.

The naive OR cannot be interpreted as a causal effect but only as a measure of association because we hypothesize that physicians allocate Cox-2 treatment based on unobserved factors that could be associated with the risk of gastrointestinal bleeding. Hence, we use SMMs in order to estimate causal effects among those treated with Cox-2 inhibitors. To recap, for a specific SMM, we know that its associated NEM assumption is required for identification of the causal effect, but in Section 4, we showed that it does not always hold. However, both the additive and multiplicative SMMs do identify local causal effects if physicians' treatment selection is monotonic. In this example, monotonicity corresponds to the assumption that no physicians who prescribe Cox-2 for patients after prescribing NSAID for their previous patients (*X*(0) = 1) would have prescribed NSAID for the same patients had they instead (counterfactually) prescribed Cox-2 to their previous patients (*X*(1) = 0). As such, unless we know that the additive NEM or monotonicity assumption holds, we cannot know if the estimate based on the additive SMM 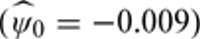 can be interpreted as the average treatment effect among the treated, or as the local treatment effect. However, the effect itself is clearly indicating less risk of gastrointestinal bleeding as the CI excludes 0.

The same scenario holds for the multiplicative SMM, but here 
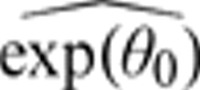
 = −0.176 and so is out of the valid range for a risk ratio. Out of range estimates are not uncommon for moment-based estimators like these. If the multiplicative NEM assumption holds, then this could be due to sampling variability: although the sample size is large, the gastrointestinal bleeding event is rare (fewer than 250 patients have events) and is sensitive to sampling variability. Alternatively, if the multiplicative NEM assumption has failed, then the negative risk ratio may indicate a failure of the monotonicity assumption. The estimate again indicates a positive effect of Cox-2 inhibitors because the CI does not include one.

The double-logistic SMM estimate (denoted DL SMM in Table 1 and using the saturated association model described in Section 2.3) is 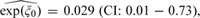 , which again indicates a positive effect of Cox-2 inhibitors. Inferences can be made about exp(*ξ*_0_) only if the logistic NEM assumption holds. If one is not prepared to believe that the logistic NEM assumption is even approximately correct, an alternative is to assume monotonicity and use the LOR estimator from Section 5. Here, it is estimated to be − 0.174, which is very close to the estimate for the multiplicative SMM and so again out of range. (Note that we would expect these estimates to be close because the gastrointestinal bleeding event is rare and so the OR approximates the risk ratio closely.) In this example, the out of range estimate again raises some doubt as to whether treatment selection is monotonic; a more likely explanation, perhaps, is that the logistic NEM assumption approximately holds and the double-logistic SMM estimate can be interpreted as evidence of a substantial positive effect of Cox-2 inhibitors among the patients to which it was allocated. The inherent problem is that these questions cannot be answered on the basis of the available data. Thus, this should be interpreted as a sensitivity analysis in which we find some degree of robustness because a positive effect of Cox-2 is inferred using all the causal estimators; in addition, we know that previously conducted randomized controlled trials have also found positive effects of Cox-2 (Brookhart *and others*, 2006).

To demonstrate further the important role of NEM, we now conduct two Monte Carlo simulation studies in which the true structural models generating the data are known. The aim of both studies is to show the impact of misinterpreting inferences obtained using SMMs.

### 6.2 Bivariate probit model simulation

The first illustration is based on structural model (4.1) from Section 3, namely,
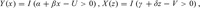
where here we set (*U*,*V*) to have the bivariate normal distribution
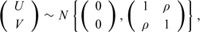
and Pr(*Z* = 1) = 0.5. Note that *ρ* indexes the strength of nonignorability in the selection mechanism determining compliance, with *ρ* = 0 corresponding to ignorable compliance. For each set of parameter values (*α*,*β*,*γ*,*δ*,*ρ*), we can calculate the corresponding values of the key causal parameters. We fix the parameters in the outcome model to *α* = 0, *β* = 0.1 and look at how the causal parameters vary as a function of (*γ*,*δ*,*ρ*).

**Fig. 1.**
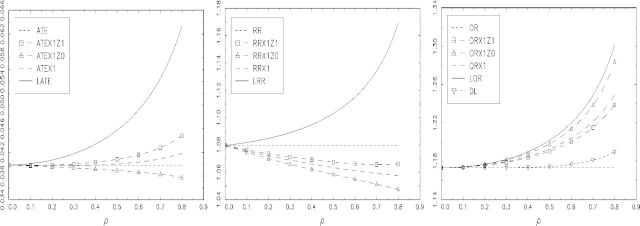
A comparison of relevant population causal parameters for data generated by randomisation indicator *Z* and the bivariate probit model defined in Section 6.2 with *α* = 0,*β* = 0.1,*γ* = 0, *δ* = 0.500: (left to right) for the additive SMM; the multiplicative SMM; and the logistic SMM. The NEM assumption does not hold for any of these SMMs but selection is monotonic.

Figure 1 displays the values of average treatment effects, RRs, and ORs as a function of *ρ* for *γ* = 0 and *δ* = 0.5. In the first panel, ATE denotes the average treatment effect *E*{*Y*(1) − *Y*(0)}, and the parameters of the additive SMM are denoted as follows: *ψ*_0_ + *ψ*_1_ = *E*{*Y*(1) − *Y*(0)|*X* = 1,*Z* = 1} by ATEX1Z1, *ψ*_0_ = *E*{*Y*(1) − *Y*(0)|*X* = 1,*Z* = 0} by ATEX1Z0, and the average treatment effect among the treated *E*{*Y*(1) − *Y*(0)|*X* = 1} by ATEX1; LATE denotes *E*{*Y*(1) − *Y*(0)|*X*(1) > *X*(0)}. The parameters are similarly defined in the second and third panel for the RR and OR, respectively (RRX1Z1 = exp(*θ*_0_ + *θ*_1_), ORX1Z0 = exp(*ξ*_0_), LRR, etc.); for the OR, there is an additional parameter, denoted by DL, corresponding to the estimand of the double-logistic SMM (2.6).

For *α* = 0 and *β* = 0.1, the marginal expectations are *E*{*Y*(1)} = Φ(0.1) = 0.540 and *E*{*Y*(0)} = Φ(0) = 0.5, and so ATE = 0.540 − 0.5 = 0.040, RR = 1.080 and OR = 1.173. Likewise, as *γ* = 0 and *δ* = 0.5 then *E*{*X*(1)} = Φ(0.5) = 0.692 and *E*{*X*(0)} = Φ(0) = 0.5, indicating a large degree of noncompliance in the control arm. The proportion of compliers in the population is Pr{*X*(1) > *X*(0)} = *E*{*X*(1) − *X*(0)} = 0.192.

Figure 1 shows the differences between the local parameters that are identified by the SMM estimands, LATE and LRR, and the respective parameters for each in the treated group, ATEX1 and RRX1. Clearly, the differences are increasing functions of *ρ*. We take ATEX1 and RRX1 as the comparison here because these are the SMM estimands if the corresponding NEM assumptions hold. The differences are quite substantial for large *ρ* : for example, if *ρ* = 0.5 then LATE equals 0.046 and ATEX1 is equal to 0.040, a difference of 14%. In terms of risk ratios, LRR minus 1 equals 0.063 and RRX1 minus 1 equals 0.103, a 62% difference. The magnitude by which NEM is violated is indicated by the difference between ATEX1Z1 and ATEX1Z0 for the additive SMM, and between RRX1Z1 and RRX1Z0 for the multiplicative SMM. Both are relatively small indicating a minor failure of NEM, but the local parameters take quite different values. For the odds ratio, LOR and ORX1 are quite close: for example, if *ρ* = 0.5 then LOR minus 1 equals 0.202 and ORX1 minus 1 equals 0.193, a small difference of only 4.8%. Interestingly, the estimand of the double-logistic SMM estimator, DL, tracks OR quite closely here, but not LOR or ORX1: at *ρ* = 0.5, DL minus 1 equals 0.175, a 10% difference from ORX1 and a 15.6% difference from LOR.

**Fig. 2.**
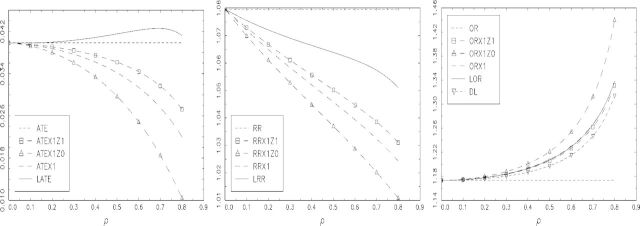
A comparison of relevant population causal parameters for data generated by randomization indicator *Z* and the bivariate probit model defined in Section 6.2 with *α* = 0,*β* = 0.1,*γ* = − 1, *δ* = 0.615: (left to right) for the additive SMM; the multiplicative SMM; and the logistic SMM. The compliance rate is higher among the controls than in Figure 2. The NEM assumption does not hold for any of these SMMs but selection is monotonic.

Figure 2 displays the same plots for *γ* = − 1 and *δ* = 0.615. We now have *E*{*X*(0)} = 0.159, so there is more compliance in the control group, while the complier proportion remains 0.192. Here, we find values of LATE and ATEX1 at *ρ* = 0.5 of 0.042 and 0.034, respectively, a difference of 21%. For the LRR and RRX1 (minus 1), the respective values are 0.064 and 0.046, a difference of 40%. In contrast, the LOR and ORX1 are virtually identical in this case for all *ρ*, with DL now tracking both quite closely.

**Fig. 3.**
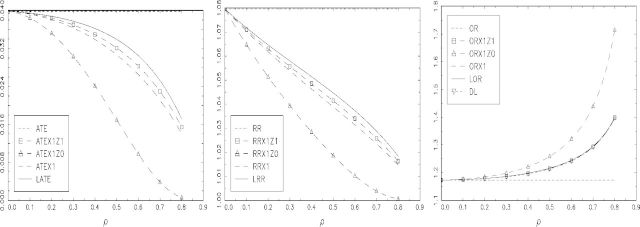
A comparison of relevant population causal parameters for data generated by randomization indicator *Z* and the bivariate probit model defined in Section 6.2 with *α* = 0,*β* = 0.1,*γ* = − 2, *δ* = 1.208: (left to right) for the additive SMM; the multiplicative SMM; and the logistic SMM. The compliance rate is very small among controls and so approximates a no contamination restriction.

In Figure 3, we set *γ* = − 1 and *δ* = 1.208 to give *E*{*X*(0)} = 0.023. These parameter values generate data for which no contamination might be expected to provide a good approximation. As expected, the local parameters LATE and LRR are very close to ATEX1Z1 and RRX1Z1, respectively, and to ATEX1 and RRX1 too. The LOR and DL are in this case identical to ORX1Z1 and ORX1.

### 6.3 Mixed logistic model simulation

Didelez *and others* (2010) consider a more complex logistic structural model for generating nonignorable noncompliance. Using our notation, this model is written(6.1)
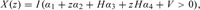

(6.2)
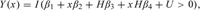
where *U* and *V* are independent standard logistically distributed random variables, and *H* is unobserved. Equivalent expressions to (6.1–6.2) are *E*{*X*(*z*)|*H* = *h*} = expit(*α*_1_ + *z* *α*_2_ + *h* *α*_3_ + *z* *h* *α*_4_) and *E*{*Y*(*x*)|*H* = *h*} = expit(*β*_1_ + *x* *β*_2_ + *h* *β*_3_ + *x* *h* *β*_4_), respectively. Both models contain interaction terms allowing the effect of latent *H* to vary depending on *z* and *x*. There are heterogeneous treatment effects on the latent scale if *β*_4_≠0, but this poses no problems as SMMs do not constrain treatment effects to be homogeneous, or indeed place any constraints on the form of treatment effect heterogeneity. More importantly, however, the monotonicity assumption *X*(1) ≥ *X*(0) holds only if *α*_4_ = 0, and monotonicity is crucial for identification of local effects.

We generate data according to models (6.1) and (6.2), setting the parameters *α*_1_ = 0, *α*_2_ = 0.5, *α*_3_ = 2, *β*_1_ = 0, *β*_2_ = 0.3, *β*_3_ = 2, and specifying *H*∼*N*(0,1) and *P*(*Z* = 1) = 0.5. Table 2 contains Monte Carlo estimates, based on 1000 replications. For the additive and multiplicative SMMs, we use (2.4) and (2.5). We further present estimation results for the consistent estimator of LOR described in Section 5 and for the double-logistic SMM, DL SMM. To minimize the impact of finite sample bias and maintain our focus on consistency, we generated samples of size 500000. The population parameters ATE, ATEX1, etc. are defined as above, but are here calculated to a high order of approximation by averaging over 1000 replicated data sets. The column denoted SD contains the Monte Carlo standard deviations of the estimators.

**Table 2.** A comparison of relevant population causal parameters together with the estimands for each SMM and local effect estimator; the data are generated by randomisation indicator Z and the mixed logistic model defined in Section 6.3 with α_1_ = 0, α_2_ = 0.5, β_1_ = 0, β_2_ = 0.3, β_3_ = 2: (1) for α_4_ = β_4_ = 0 selection is monotonic and the treatment effect is constant on the logistic scale; (2) for α_4_ = 1, β_4_ = 0 the treatment effect is heterogeneous; and (3) for α_4_ = β_4_ = 1 selection is nonmonotonic

	(1)		(2)		(3)	
	*α*_4_ = *β*_4_ = 0		*α*_4_ = 0, *β*_4_ = 1		*α*_4_ = 1, *β*_4_ = 1	
	Mean	SD	Mean	SD	Mean	SD
ATE	0.045		0.034		0.034	
ATEX1Z1	0.046		0.062		0.071	
ATEX1Z0	0.044		0.066		0.066	
ATEX1	0.045		0.064		0.068	
LATE	0.057		0.040		0.092	
Add. SMM	0.057	0.0179	0.040	0.0181	0.114	0.023
RR	1.091		1.069		1.069	
RRX1Z1	1.068		1.094		1.101	
RRX1Z0	1.063		1.094		1.094	
RRX1	1.066		1.094		1.098	
LRR	1.122		1.085		1.138	
Mult. SMM	1.122	0.040	1.086	0.040	1.152	0.033
OR	1.199		1.148		1.148	
ORX1Z1	1.238		1.346		1.445	
ORX1Z0	1.242		1.398		1.398	
ORX1	1.239		1.369		1.423	
LOR	1.259		1.175		1.580	
LOR estimator	1.261	0.090	1.178	0.085	2.214	0.366
DL SMM	1.220	0.077	1.142	0.069	2.039	0.261

1000 Monte Carlo replications of sample size 500 000; the population parameters were calculated by averaging over each generated data set.

The results for *α*_4_ = *β*_4_ = 0 are given in column 1 and are similar to the results found in the first example above. When we introduce an extra source of treatment heterogeneity by setting *β*_4_ = 1 (column 2), we see again that the additive, multiplicative, and LOR SMM estimators are very close to the local parameters. For the OR, it can also be seen that treatment effect heterogeneity has here exacerbated the difference between the local and treated group ORs, LOR and ORX1 being 1.175 and 1.369 respectively; the double-logistic SMM estimator DL SMM is close to the OR in this case.

When the monotonicity assumption is violated by further setting *α*_4_ = 1 (column 3), we see that the three SMM estimates diverge from the local parameters, with the LOR estimator especially poorly behaved. In this example, the divergence between the target parameters, the causal effects in the treated group, and the estimates of the local treatment effects gets more pronounced. The DL SMM estimator overestimates all of the causal treatment effect parameters.

## 7. CONCLUSIONS

We have highlighted that causal effects on binary outcomes in studies with nonignorable non-compliance are not always identified by SMMs. Additional assumptions about the causal process generating the observed data are required, but in practice these are difficult to verify. The simulation study examples above show that failure of these assumptions can lead to misleading inferences.

An exception to this rule is when the study design satisfies the no contamination restriction that the control group has no access to treatment (e.g., randomized placebo-controlled trials). However, SMMs are now being applied to observational studies where *Z* is an instrumental variable (Vansteelandt *and others*, 2010), including applications using genetic instruments that exploit the “Mendelian randomization” hypothesis (e.g., Didelez and Sheehan, 2007). For these designs, a no contamination restriction is unlikely to hold, as was the case in our data example.

An alternative assumption is to assume that the mechanism by which patients select treatment is monotonic. Under monotonicity, the additive and multiplicative SMM estimators are valid for local causal effects, but these can be quite different from treatment effects for the treated. Caution is therefore required when interpreting SMM estimates for binary outcomes if patients in the control group can receive treatment, with the issues of monotonicity and the interpretation of local/complier average effects paramount. When the NEM assumption fails, we find that the double-logistic estimator is not consistent for the LOR under monotonicity, but point out that an alternative estimator is available that is consistent.

If the practitioner is agnostic about any of the above as reasonable working assumptions, then we would recommend he/she performs a sensitivity analysis. In addition to the SMMs discussed here, various causal estimators for binary outcomes based on instrumental variable estimators have been proposed in the literature; see reviews by Vansteelandt *and others* (2010), Clarke and Windmeijer (2009) and Didelez *and others* (2010). Each estimator makes alternative identifying assumptions, and assessing robustness to these different assumptions should be regarded as essential.

Finally, we note that recent work by van der Laan *and others* (2007) proposes alternative modelling strategies to those developed by Vansteelandt and Goetghebeur (2003) and Robins and Rotnitzky (2004), but this approach is not yet widely used.

## FUNDING

UK Economic & Social Research Council (RES-060-23-0011); UK Medical Research Council (G0601625).
